# Hospital admissions in older people with visual impairment in Britain

**DOI:** 10.1186/1471-2415-8-16

**Published:** 2008-09-11

**Authors:** Jennifer R Evans, Liam Smeeth, Astrid E Fletcher

**Affiliations:** 1 International Centre for Eye Health, London School of Hygiene and Tropical Medicine (LSHTM), Keppel Street, London, WC1E 7HT, UK; 2 Non-Communicable Disease Epidemiology Unit, LSHTM, Keppel Street, London, WC1E 7HT, UK

## Abstract

**Background:**

We aimed to assess the risk of hospital admission associated with visual impairment in a representative sample of older people living in the community in Britain.

**Methods:**

Design: Prospective study of hospital admission in a population-based sample of community dwelling people aged 75 years and above in Britain. Setting: 53 general practices. Participants: 14,394 participants in the MRC Trial of Assessment and Management of Older people in the Community. Main outcome measure: Hospital admission.

**Results:**

Visually impaired older people had 238.7 admissions/1000 person-years compared to 169.7 admissions/1000 person-years in people with good vision: age and sex adjusted rate ratio (RR) 1.32 (95% CI 1.19 to 1.47). Adjusting for a wide range of potential explanatory factors largely eliminated this association: RR 1.06 (95% CI 0.94 to 1.20). However, adjusting for a more limited range of confounding factors, excluding those factors possibly a consequence of reduced vision, left a modest increased risk: RR 1.19 (95% CI 1.06 to 1.34).

**Conclusion:**

The association between visual impairment and rate of hospital admission can be attributed to higher levels of co-morbidity and reduced functional ability among people with reduced vision. Visual impairment is likely to be an important contributor to reduced functional ability, but other factors may also be involved.

## Background

The prevalence of visual impairment in older people admitted to hospital has been estimated to be as high as 50%[1]. There is limited evidence as to the risk of hospital admission in visually impaired people. One study suggested that there is an increased risk of hospital admission in visually impaired people[2] and one study that visual impairment increases the average length of stay in hospital[3].

The aim of this study was to assess the risk of hospital admission associated with visual impairment in a representative sample of older people living in the community in Britain.

## Methods

The Medical Research Council (MRC) trial of the assessment and management of older people in the community was a large cluster randomised trial in 106 general practices from the MRC General Practice Research Framework[4]. The practices in the study were selected to be representative of the mortality (SMR) and Jarman scores of general practices in Britain (England, Wales and Scotland). The aim of the trial was to evaluate the benefit of different methods of assessment and management of older people in the context of the 1990 contract of service which required general practitioners in the UK to offer an annual health check to patients aged 75 years and over. The main results of the trial have been published[5]. The study compared two different types of multidimensional assessment (targeted versus universal) and two different management models (primary care team versus multidisciplinary geriatric evaluation team). Randomisation was at the practice level and stratified by standardised mortality ratio and Jarman score. All patients aged 75 years or over on the general practitioner list were invited to participate in the trial, unless they were in long stay hospital or nursing homes, or were terminally ill.

People in the 53 practices allocated to the "universal" arm of the trial were given a visual acuity test as part of a detailed health assessment by the practice nurse. Visual acuity was measured at 3 metres with a Glasgow Acuity Chart which measures the minimal angle of resolution on a logarithmic scale[6]. Vision was measured both as presenting vision (with spectacle or contact lenses) and also in each eye. People with binocular presenting vision better than 6/9 were defined as "good vision", those with presenting vision less than 6/9 to 6/18 as reduced vision. Visual impairment was defined as presenting binocular acuity of less than 6/18 (logMAR score 0.5 or more). In 49 practices, the cause of visual impairment was assessed by medical record review[7]. Information on hospital admission was collected by trial nurses on an ongoing basis for each patient from the date of the baseline assessment. The baseline assessments were conducted during four years 1995 to 1998; hospital admissions were collected by study nurses for each patient for a 2-year period from baseline (invitation to assessment) from hospital discharge letters in the patients' GP records. The definition of a hospital admission included an overnight stay. The trial and additional data collection on the cause of visual loss was approved by the relevant local research ethics committees.

All analyses were done using Stata version 10.0 and took into account the cluster design of the study using "svy" commands (Stata Corporation, College Station, Texas 77845, USA). People with an MMSE score of less than 12 were excluded from these analyses because measurement of visual acuity in this group is likely to be inaccurate. The rate ratio of hospital admission associated with visual impairment was estimated using poisson regression. In all analyses the reference group was those with "good vision". The following potential confounding factors and effect modifiers were considered: age, sex, marital status (single/married/widowed), living alone, housing tenure (home owner/not home owner/sheltered accommodation), financial difficulties (difficulties making ends meet and/or managing finances), looked after someone with a serious illness in the last year, death of loved one in last year, social support (no relative/friend to call on and/or no help at night), alcohol consumption (never/ex/current below median/current above median), smoking (never/ex/current), body mass index (BMI) (quintiles), depression (6 or more on Geriatric Depression Scale), diabetes, hearing impairment (failed whispered voice test), reported major illness (heart attack, stroke, Parkinsons disease, cancer), self-reported health (excellent or very good/good/fair or poor), activities of daily living (ADL) score (unable to do 0–1 ADLs, unable to do 2–4 ADLs, unable to do 5–8 ADLs), falls in last six months (none/one/two or more), self-reported activity (very or fairly/not very or not at all), cognitive impairment (Mini Mental State Examination (MMSE) score 12-17/18-23/24-30).

Only those variables statistically associated with both hospital admission and visual impairment in this dataset (after controlling for age and sex) were considered as potential explanatory factors in addition to age and sex. These were: marital status, housing tenure, financial difficulties, alcohol consumption, BMI, depression, diabetes, reported major illness, self-reported health, falls, ADL, self-reported activity, MMSE. Because vision impairment may lead to problems with self-reported health, activity, ADLs, falls and depression, and therefore these could be considered to be on the "causal pathway" and thus be explanatory factors rather than confounders, analyses were carried out with and without these variables.

## Results

Response rates and characteristics of non-responders have been published elsewhere[7]. 15,336/21,762(70%) of eligible people in practices randomised to the universal arm of the MRC trial had a detailed assessment. People taking part were slightly younger and more likely to be men compared to those who did not take part. 14,394/15,336 of people with a detailed assessment were included in the current analyses. Reasons for exclusion were: MMSE < 12 (n = 228), or no data on vision (n = 484), data on baseline/follow-up not available (n = 230). Table 1 shows the characteristics of the study population.

**Table 1:** Characteristics of study population

	Binocular presenting vision with usual glasses				
			
	Good vision (better than 6/9) N = 7107	Reduced vision (6/9 to 6/18) N = 5564	Visually impaired (worse than 6/18) N = 1723	Missing data	Association with visual impairment, after adjusting for age and sex*
Median binocular acuity (*IQR range*)**	0.025 (-0.025,0.1)	0.3 (0.225,0.35)	0.625 (0.525, 0.8)	167	NA
Mean age(*SD*)	79.7 (3.8)	82.0 (4.7)	84.3 (5.4)	66	p < 0.001
90 years and older *(N%)*	115(1.6)	362(6.5)	258(15.0)	66	p < 0.001
Female *(N%)*	3997(56.2)	3602(64.7)	1220(70.8)	0	p < 0.001
Married *(N%)*	3419(49.2)	2037(37.4)	477(28.2)	291	p = 0.005
Widowed *(N%)*	3148(45.3)	3068(56.3)	1074(63.4)		
Lives alone *(N%)*	3055(43.0)	2827(50.8)	936(54.3)	0	p = 0.293
Home owner *(N%)*	4872(68.9)	3342(60.4)	929(54.8)	97	p < 0.001
Sheltered accommodation *(N%)*	427(6.0)	527(9.5)	233(13.7)		
Financial difficulties *(N%)*	294(4.2)	344(6.3)	200(11.9)	189	p < 0.001
Looked after someone with a serious illness in last year *(N%)*	963(13.8)	727(13.3)	215(12.8)	255	p = 0.891
Death of loved one in last year *(N%)*	1168(16.6)	922(16.8)	317(18.7)	157	p = 0.117
No relative/friend to call on/no help at night *(N%)*	436(6.5)	346(6.8)	100(6.6)	1071	p = 0.915
Drinks above median alcohol consumption *(N%)*	1519(21.5)	982(17.8)	250(14.7)	117	p < 0.001
Current smoker *(N%)*	679(9.6)	561(10.1)	204(11.9)	22	p < 0.001
Mean body mass index (BMI)*Mean (SD)*	26.2 (4.1)	26.0 (4.3)	25.3 (4.5)	1157	p = 0.010
Lightest fifth for BMI *(N%)*	1209(17.9)	1009(20.0)	410(28.5)		p = 0.018
Depression (N%)**	378(5.4)	493(9.1)	219(13.2)	381	p < 0.001
Diabetes *(N%)*	500(7.0)	448(8.1)	164(9.5)	0	p < 0.001
% Hearing impairment *(N%)*	1443(20.5)	1495(27.2)	613(36.1)	156	p = 0.002
Reported major illness *(N%)*	1898(26.7)	1750(31.5)	585(34.0)	20	p < 0.001
Reported fair/poor health *(N%)*	837(11.8)	988(17.9)	394(23.1)	88	p < 0.001
Unable to do 2–4 ADL *(N%)*	1002(14.2)	1300(23.6)	610(36.0)	120	p < 0.001
Unable to do 5–8 ADL *(N%)*	307(4.4)	626(11.4)	394(23.2)		
2 or more falls in last six months *(N%)*	408(5.8)	543(9.8)	221(13.0)	53	p = 0.005
Not very/not at all active *(N%)*	1128(15.9)	1456(26.3)	640(37.7)	87	p < 0.001
MMSE 12–17 *(N%)*	173(2.5)	252(4.6)	225(13.3)	138	p < 0.001
MMSE 18–23 *(N%)*	800(11.3)	1051(19.1)	542(32.1)		

IQR = Interquartile range; ADL activities of daily living; MMSE mini mental state examination score.

* From multinomial logistic regression model. Dependent variable visual impairment (3 categories). Independent variables age, sex and row variable. All tests take into account the cluster design of the study.

**LogMAR score

Table 2 shows the rate of hospital admission in visually impaired people. The rate of hospital admission in visually impaired people was 238.7/1000 person-years compared to 169.7/1000 person-years in people with good vision. The rate ratio adjusted for age and sex was 1.32 (95% CI 1.19 to 1.47). Adjusting for a wide range of potential explanatory social and co-morbidity factors eliminated this increased risk of hospital admission in visually impaired people. The rate ratio adjusting for all factors was 1.06 (95% CI 0.94 to 1.20). We repeated the analyses excluding variables potentially on the causal pathway. The rate ratio for hospital admission was 1.19 (95% CI 1.06 to 1.34).

**Table 2:** Hospital admission in visually impaired people aged 75 years and above

				Model 1		Model 2		Model 3	
						
Binocular presenting vision with usual glasses.	Number of people with data on hospital admission	Admissions	Person-years	Rate ratio	95% CI	Rate ratio	95% CI	Rate ratio	95% CI
Good vision > 6/9	7107	2302	13561.9	1		1		1	
Reduced vision 6/9 to 6/18	5564	2215	10329.3	1.22	1.12,1.34	1.10	1.00,1.21	1.17	1.07,1.28
Visually impaired < 6/18	1723	737	3087.3	1.32	1.19,1.47	1.06	0.94,1.20	1.19	1.06,1.34

Total	14394	5254	26978.6	N in model = 14328		N in model = 13494		N in model = 13940	

CI: confidence intervals MMSE: mini-mental state examination

53 practices; people with MMSE score of less than 12 were excluded; analyses take into account the cluster design of the study

Model 1: adjusted age and sex

Model 2: adjusted age, sex, housing tenure, financial difficulties, smoking, depression, diabetes, reported major illness, self-reported health, falls, ADL, self-reported activity, MMSE.

Model 3: adjusted age, sex, housing tenure, financial difficulties, smoking, diabetes, reported major illness, MMSE.

Table 3 shows the rate of hospital admission by cause of visual impairment. After adjustment for confounders there were no significant associations between cause of vision impairment and risk of admission.

**Table 3:** Association between cause of visual impairment and hospital admission

	Model 1		Model 2		Model 3	
			
	Rate ratio	95% CI	Rate ratio	95% CI	Rate ratio	95% CI
Visual acuity 6/9 or better	1		1		1	
AMD	1.32	0.98,1.79	0.96	0.70,1.32	1.13	0.83,1.54
Cataract	1.46	1.09,1.96	1.20	0.86,1.67	1.31	0.96,1.78
Other causes	1.42	1.02,1.97	1.11	0.74,1.67	1.28	0.89,1.86
Refractive error	1.31	1.04,1.66	1.13	0.87,1.45	1.20	0.94,1.54
Cause unknown	1.28	0.89,1.83	1.14	0.78,1.68	1.18	0.82,1.70

CI: confidence intervals

49 practices; people with MMSE score of less than 12 were excluded; analyses take into account the cluster design of the study

Model 1: adjusted age and sex

Model 2: adjusted age, sex, housing tenure, financial difficulties, smoking, depression, diabetes, reported major illness, self-reported health, falls, ADL, self-reported activity, MMSE.

Model 3: adjusted age, sex, housing tenure, financial difficulties, smoking, diabetes, reported major illness, MMSE.

The reason for hospital admission was specified for 87% of admissions, i.e. the speciality of the admission was recorded. 1.5% of admissions were classified as "accident and emergency". However, this figure probably represents admissions to small short stay wards found in some (but not all) casualty departments and is unlikely to reflect true visits to emergency departments. There was little association between visual impairment and risk of an "accident and emergency" admission (risk ratio controlling for all explanatory factors 0.73, 95% CI 0.29 to 1.84).

There were 303 admissions recorded as "ophthalmology". Unsurprisingly people with visual impairment were more likely to be admitted for one or more ophthalmology admissions (risk ratio controlling for all explanatory factors 3.10, 95% CI 2.24 to 4.29). Excluding these ophthalmology admissions did not change the results: the rate ratio adjusted for age and sex was 1.31 (95% CI 1.17 to 1.46); the rate ratio adjusting for all factors was 1.04 (95% CI 0.92 to 1.18); and the rate ratio adjusted for variables not potentially on the causal pathway was 1.17 (95% CI 1.04 to 1.32).

Visually impaired people had a longer median duration of hospital stay compared to people with good vision (12 days vs 8 days), p < 0.001 Wilcoxon rank sum test. After adjustment for explanatory factors not on the causal pathway visually impaired people stayed on average two extra days in hospital (p = 0.408).

## Discussion

We found some evidence for an increased risk of hospital admission in visually impaired older people in the MRC trial over two years follow-up. Part of this increased risk was attributable to higher levels of co-morbidity and part attributable to factors which may be the consequence of reduced vision, such as reduced functional ability. However, in this large epidemiological study we cannot be sure for each individual exactly what caused the reported reduced functional ability – whether it was the visual impairment or some other factor such as arthritis or cardiovascular disease. Visual impairment and difficulty with activities of daily living were associated after controlling for all other factors which suggests that part of this reduced functional ability could be attributable to visual loss. However, analyses of the cause of visual impairment and hospital admission found no increased risk of hospital admission for causes of visual loss such as age-related macular degeneration. If the increased risk of hospital admission was due to the visual impairment we might expect that conditions such as age-related macular degeneration which are associated with more severe visual impairment might show the effect more strongly. However this was not the case.

There are not many other studies on this topic. Jack et al measured the prevalence of visual impairment in 200 people aged 65 years and above admitted to one UK hospital with an acute medical illness[1]. They found that half of these people were visually impaired. Although this is a high prevalence of visual impairment it is difficult to interpret as the age structure of the hospital admissions is unlikely to reflect population age structure and the prevalence of visual impairment increases rapidly over the ages of 65 years and above[8,9]. Jacobs et al studied 839 people aged 77 in Israel and found that visual impairment was associated with an increased likelihood of attending for emergency care in men but not in women[2]. However, these analyses were not controlled for age or any other potentially confounding factors. Morse et al in New York State, USA, analysed routinely collected data on length of hospital stay[3]. Visual impairment was associated with an extra two days length of stay in hospital, after controlling for age, sex, payer source and other co-morbid conditions. We similarly found a non-significant increased length of stay in visually impaired older people of two days, after adjusting for explanatory factors not on the causal pathway.

Although these associations are interesting it cannot be assumed that screening for visual impairment in the community would necessarily lead to reductions in hospital admission and length of stay. Screening for visual impairment in this age-group does not appear to change the prevalence of visual impairment in this age-group[10]. The MRC trial which evaluated the benefit of screening for a wide range of health and social problems including vision did not find a benefit on rates of hospital admission[5].

## Conclusion

The association between visual impairment and rate of hospital admission can be attributed to higher levels of co-morbidity and reduced functional ability among people with reduced vision. Visual impairment is likely to be an important contributor to reduced functional ability, but other factors will also be involved. This underlines the importance of taking a wider view of an older person rather than focussing on a single impairment or disability.

## Competing interests

The authors declare that they have no competing interests.

## Authors' contributions

JRE contributed to the conception and design of the study, collected data on cause of visual loss, did the analyses and drafted the paper. AF and LS contributed to the conception and design of the study, interpretation of the results and critical revision of the manuscript for important intellectual content. All authors read and approved the final manuscript.

## Pre-publication history

The pre-publication history for this paper can be accessed here:


